# Does GP training in depression care affect patient outcome? - A systematic review and meta-analysis

**DOI:** 10.1186/1472-6963-12-10

**Published:** 2012-01-10

**Authors:** Claudia Sikorski, Melanie Luppa, Hans-Helmut König, Hendrik van den Bussche, Steffi G Riedel-Heller

**Affiliations:** 1 Institute of Social Medicine, Occupational Health and Public Health, University of Leipzig, Leipzig, Germany; 2 IFB AdiposityDiseases, Leipzig University Medical Center, Leipzig, Germany; 3 Department of Medical Sociology and Health Economics, Hamburg-Eppendorf University Medical Center, Hamburg, Germany; 4 Department of Primary Medical Care, Hamburg-Eppendorf University Medical Center, Hamburg, Germany; 5 Institute of Social Medicine, Occupational Health and Public Health, University of Leipzig, Leipzig, Germany

**Keywords:** depression, primary care, training, health service

## Abstract

**Background:**

Primary care practices provide a gate-keeping function in many health care systems. Since depressive disorders are highly prevalent in primary care settings, reliable detection and diagnoses are a first step to enhance depression care for patients. Provider training is a self-evident approach to enhance detection, diagnoses and treatment options and might even lead to improved patient outcomes.

**Methods:**

A systematic literature search was conducted reviewing research studies providing training of general practitioners, published from 1999 until May 2011, available on the electronic databases Medline, Web of Science, PsycINFO and the Cochrane Library as well as national guidelines and health technology assessments (HTA).

**Results:**

108 articles were fully assessed and 11 articles met the inclusion criteria and were included. Training of providers alone (even in a specific interventional method) did not result in improved patient outcomes. The additional implementation of guidelines and the use of more complex interventions in primary care yield a significant reduction in depressive symptomatology. The number of studies examining sole provider training is limited, and studies include different patient samples (new on-set cases vs. chronically depressed patients), which reduce comparability.

**Conclusions:**

This is the first overview of randomized controlled trials introducing GP training for depression care. Provider training by itself does not seem to improve depression care; however, if combined with additional guidelines implementation, results are promising for new-onset depression patient samples. Additional organizational structure changes in form of collaborative care models are more likely to show effects on depression care.

## Background

Depressive disorders are highly prevalent in the general public. The 12-month prevalence of Major Depression among Europeans has shown to be approximately 6.9% while conservative estimates of the lifetime prevalence range up to 14% [1,2]. Depression is associated with significant functional impairment and reduced quality of life [3,4], excess mortality rates [5] and particularly high costs for society and health care systems [6-9]. Considering the large effects of the disease on individuals and society, it seems clear that early detection and treatment is a desirable goal in order to promote remission and reduce negative consequences [10].

While 50 to 70 per cent of all depressed patients consult their primary care physician during an episode, therefore making them the profession most likely to be seen [11,12], depression in primary care remains under-recognised and under-treated [13]. As Bijl and colleagues (2004) summarise, the elements of detection, diagnosis and treatment determine successful depression management in health care. Previous trials showed that approximately 50 per cent of all depressed patients in primary care were not diagnosed as such [14-16]. This is a major downfall in depression care, since even subthreshold depression episodes may be clinically significant [17]. Obviously part of this is due to reluctance to seek treatment among patients themselves, resulting from concerns on effectiveness of treatment and perceived absence of treatment necessity [18].

To objectify diagnoses, the use of screening instruments has been promoted by the U.S. Preventive Services Task Force, when adequate treatment and care possibilities are available [19]. Reviews showed that screening alone does not improve depression outcomes for patients [20], but needs further organizational changes [21]. These structural interventions, including collaborative care approaches as well as provider training, represent an attempt to increase detection and diagnosis of depressed patients and therefore promote enhanced treatment. Several treatment options that support primary care physicians in treatment and that are also directly delivered by general practitioners (such as PST- problem solving therapy) have been found to be effective for depression [22,23].

Primary care practices play a central role in many health care systems- this kind of gate-keeping is even associated with improved coordination and outcomes [24]. This circumstance makes general practitioners ideal as a base for first steps in treatment, also referred to as a "stepped care" approach [25]. "Watchful waiting" and low intense interventions such as self-help approaches have to be encouraged as useful strategies [26]. In order to make full use of this opportunity, improvement of detection rates and diagnosis is inevitable. Improving skills of primary care providers can be achieved by different strategies. Consultation-liaison involves a persistent educational supervision of the general practitioner by a mental health specialist. This approach has not been shown to be effective in reducing depressive symptoms [27]. As indicated by Cape et al. (2010), another point of intervention can be to train primary care providers in diagnosis and treatment strategies without the inclusion of mental health specialists (such as collaborative care), considering limited financial resources of health care systems [27]. Moderated by higher detection rates and better knowledge on treatment options, improvement on this level could subsequently lead to higher remission rates in less time and improved depression outcomes.

In the past, these programmes of provider education have been evaluated, yielding unclear results on effectiveness of the intervention regarding health gain outcomes [21]. This study therefore reviews current literature for an updated overview. It is the first overview of randomized controlled trials that exclusively investigates interventions that apply practitioner training.

## Methods

### Literature Search

This review was prepared according to the systematic literature review guidelines of the Centre for Reviews and Dissemination [28] and follows PRISMA (Preferred Reporting Items for Systematic reviews and Meta-Analyses) suggestions [29]. A systematic literature search was conducted reviewing research studies, published from 1999 until May 2011, available on the electronic databases Medline, Web of Science, PsycINFO and the Cochrane Library as well as national guidelines and health technology assessments (HTA). In addition, the bibliographies of the selected articles were searched. Grey literature was not searched. 1999 as a starting point of the search was chosen to include at least the last 10 years of publications. The latest review on this topic, including studies from 1999 onward, was conducted in 2003, and we meant to include those studies as well [21]. The aim was to evaluate if newer, more recent studies would show clearer effects of the intervention than previous overviews.

The terms (depression OR depressive disorder) AND (general practitioner OR general practice OR primary care OR family practice) AND (training OR education) served as search criteria within titles and abstracts. All terms were also used as MeSH terms where applicable. Test searches were run preceding the actual search in order to determine the right search terms. Additional File 1 shows the Medline search strategy in detail. The search was limited to English and German language publications.

### Inclusion criteria

Abstracts were screened by two authors using the following inclusion criteria: (i) randomized controlled trials (RCT) or review articles (ii) of the adult (≥ 18 years) general population, (iii) evaluating interventional programmes including general practitioner training, mentioned in the abstract and (iv) reporting effects on depressive symptomatology. All extracted review articles were scanned and hand-searched for further relevant publications from 1999 onward.

Studies examining effects in specific study samples (such as diabetic patients with co-morbid depression) were excluded. Research of those specific samples was thought to provide only limited evidence for primary health care patients in general. All articles where a clear decision could not be made based on title or abstract were retrieved for a more detailed analysis. In case of disagreement, a third reviewer was consulted and then a consensus decision was achieved.

Training and education of general practitioners was defined as a professional intervention [21] that involves the use of guidelines or short training classes with a focus on optimising diagnosis as well as treatment. Studies involving additional organisational interventions were excluded. Additional file 2 gives an overview on excluded studies.

### Data extraction

Primarily, methodical data on sampling, study design, intervention procedure, and outcome criteria were extracted from all selected studies. Extraction of results focussed on assessing symptom alteration primarily. Only data related to a change in symptom severity (scale scores, remission rates) were extracted. Effect sizes were only calculated for the outcomes considered as relevant (symptom change). Secondly, the selection criteria described in the above section were then reapplied to ensure accurate study inclusion.

### Study Quality

Study quality was also assessed by two independent raters using a modified scale based on work by Moncrieff and colleagues [30]. The scale was modified by leaving out irrelevant items such as medication side-effects. It consists of 21 items leading to a maximum score of 42 points (Table 1). Each item, if not specified otherwise, was scored as 2 (fully met the quality criterion), 1 (partially met the quality criterion) or as 0 (did not meet the quality criterion). After a first independent run-through of ratings, the two raters met with a third independent researcher in order to discuss disagreements in scoring until a consensus was reached. Study protocols were consulted where possible.

**Table 1:** Quality assessment (Based on Moncrieff et al., 2001)

Criterion	Score and rating criteria
(1) Objectives and specification main outcomes a priori	0 = objectives unclear 1 = objectives clear but main outcomes not specified a priori 2 = objectives clear with a priori specification of main method for assessment of outcome

(2) Adequate sample size (n per group)	0 = inadequate (< 50/group) 1 = moderate (50-100/group) 2 = large (> 100/group or justified by power calculations)

(3) Appropriate duration of trial including follow up	0 = too short (< 3 months) 1 = reasonable length (3-6 months) 2 = long enough for assessment of long term outcomes (6-12 months)

(4) Power calculation	0 = not reported 1 = mentioned without details 2 = details of calculations provided

(5) Method of allocation	0 = unrandomized and likely to be biased 1 = partially or quasi randomized with some bias possible 2 = randomized allocation

(6) Concealment of allocation	0 = not done or not reported 2 = concealment of allocation code detailed

(7) Clear description of treatments (including doses of drugs used) and adjunctive treatments	0 = main treatments not clearly described 1 = inadequate details of main or adjunctive treatments 2 = full details of main and adjunctive treatments

(8) Blinding of subjects	0 = not done 1 = done but no test of blind 2 = done and integrity of blind tested

(9) Source of subjects described and representative sample recruitment	0 = source of subjects not described 1 = source of subjects given but no information on sampling or use of unrepresentative sample (for example, volunteers) 2 = source of subjects described plus representative sample taken (for example, all consecutive admissions or referrals, or random sample taken)

(10) Use of diagnostic criteria (or clear specification of inclusion criteria)	0 = none 1 = diagnostic criteria or clear inclusion criteria 2 = diagnostic criteria plus specification of severity

(11) Record of exclusion criteria and number of exclusions and refusals reported	0 = criteria and number not reported 1 = criteria or number of exclusions and refusals not reported 2 = criteria and number of exclusions and refusals reported

(12) Description of sample demographics	0 = little/no information (only age/sex) 1 = basic details (for example, marital status/ethnicity) 2 = full description (for example, socioeconomic status, clinical history)

(13) Blinding of assessor	0 = not done 1 = done but no test of blind 2 = done and integrity of blind tested

(14) Record of number and reasons for withdrawal by group	0 = no info on withdrawals by group 1 = withdrawals by group reported without reason 2 = withdrawals and reason by group

(15) Outcome measures described clearly (and therefore replicable) or use of validated (or referenced) instruments	0 = main outcomes not described clearly 1 = some of main outcomes not clearly described 2 = main outcomes clearly described or valid and reliable instruments used

(16) Information on comparability and adjustment for differences in analysis	0 = no information on comparability 1 = some information on comparability with appropriate adjustment 2 = sufficient information on comparability with appropriate adjustment

(17) Inclusion of all subjects in analyses (Intention to treat analysis)	0 = no 2 = yes

(18) Presentation of results with inclusion of data forre-analysis of main outcomes (for example, SDs)	0 = little information presented 1 = adequate information 2 = comprehensive

(19) Appropriate statistical analysis (including correction for multiple tests where applicable)	0 = inadequate 1 = adequate 2 = comprehensive and appropriate

(20) Conclusions justified	0 = no 1 = partially 2 = yes

(21) Declaration of interests (for example, 0 = no source of funding)	0 = no 2 = yes

### Effect Size and meta-analysis

Whenever applicable, standardized mean effect sizes (Cohen's d) were calculated from the data reported. At times, studies only reported scores that could not be used in effect size calculation and efforts to retrieve data directly from the authors were made. Data was entered to interpret negative standardized means in favour of the intervention. Results of cluster trials were used when the authors accounted for the effect of cluster randomization properly. According to Cohen (1988), effect sizes of 0.2 are considered small, while d = 0.5 represents a moderate effect and d = 0.8 is regarded a large effect [31]. A meta-analysis was conducted using Review Manager Software [32]. Due to the diversity of GP training in the studies, standardized mean effects were pooled - firstly, for studies with GP training only, secondly, for studies introducing additional guidelines and lastly for studies including more complex interventions. Subgroup analysis (patient inclusion, age of patients) were not carried out due to the unavailability of sufficient data. Analysis of the heterogeneity of prevalence across studies was done through I^2 ^statistic and Cochran Q. A fixed effect model was applied since heterogeneity was low.

## Results

### Search results

**Figure 1:**
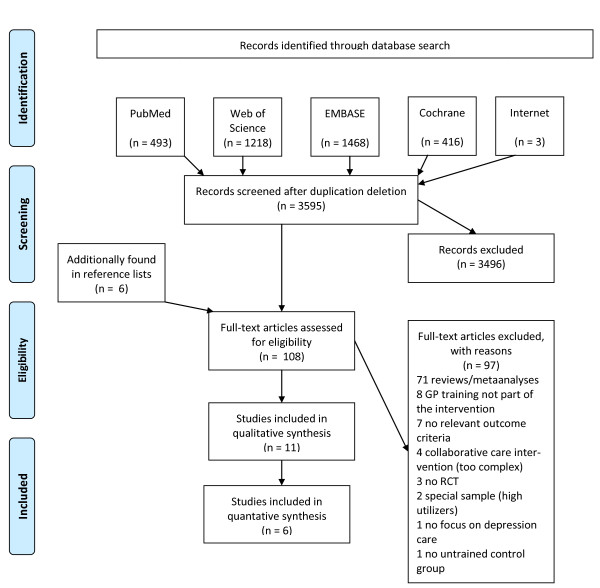
**Search Strategy**.

The results of the systematic literature search are shown in Figure 1. Interrater reliability showed substantial agreement (Kappa = 0.74). Overall, 108 potentially relevant articles were identified. After retrieving all full articles, 97 further articles were rejected as not fulfilling the selection criteria. Eleven articles were assessed and included for detailed analysis. Relevant study characteristics can be found in Table 2. Three articles are double publications of the same studies and will be subtracted for the following overview. The QuEST intervention is described in detail in a publication by Rost et al. (2000). Therefore, this reference will be used when referring to that study.

**Table 2:** Study Characteristics

Study	Country	Recruitment, Inclusion Criteria	Randomization	N^a^	Intervention, Role of GP Training	Control Group	Comparison	Quality
Baker (2001) [36]	GB	Consecutive patients; ≥ 18 yrs Patients seeking consultation for new-onset depression	Practices	402	Tailored intervention to promote guideline implementation (additional feedback, educational visits, group discussions) Additional to guideline	UC^b^	Patients of experimental group vs. control group	30

Bosmans (2006) [33]	NL	Consecutive patients; ≥ 55 yrs PRIME-MD = MD	Practices	145	4 hrs training session on screening, diagnosis and treatment as in Dutch guidelines	UC	Patients of experimental group vs. control group	39

Gask (2004) [38]	GB	GP referral; 16-65 yrs Intention or current treatment of depression (symptoms < 6 mo) HAM-D ≥ 13	Practice	189	Acquisition of clinical skills, 5 lectures à 2 hrs on assessment and treatment; Sole intervention	WL	Patients of experimental group vs. control group	36

Kendrick (2001) [34] Thompson (2000) [35]	GB	Consecutive patients; ≥ 16 yrs HADS-D ≥ 8	Practice	733	Guideline implementation & GP training (4 h seminars, educators available for 9 more mo); Additional to guideline	WL	Patients of experimental group vs. control group Sensitivity of recognition rates of experimental group vs. control group	36

King (2002) [41]	GB	Consecutive patients; ≥ 18 yrs HADS-D/A ≥ 11	Practice	272	Training of GPs in brief cognitive behaviour therapy (4 half day workshops); Sole intervention	WL	Patients of experimental group vs. control group	34

Llewellyn-Jones (1999) [40]	AUS	Residential facility; ≥ 65 yrs GDS ≥ 10 MMSE ≥ 18	Patient	220	Shared Care Intervention, including GP training & education, health education and promotion, psychoeducation; Central part of complex intervention	WL^c^	Experimental group vs. control group	33

Rost (2001) [44] Pyne (2003) [43] Rost (2005) [45]	USA	GP referral; DSM III-R MD (latter two studies exclude patients currently in treatment)	Practice	479	QuEST intervention, 4 academic telephone calls to implement guidelines, nurse w/8-hour face-to-face training; Guidelines implementation	UC	Patients of experimental group vs. control group^d^	38

Worrall (1999) [39]	CAN	GP referral; GP diagnosis and severity rating, later CES-D ≥ 16	Practice	147	3-hour sessions on guideline implementation + possible consultation of psychiatrist; Guidelines implementation	UC^b^	Patients of experimental group vs. control group	26

^a ^At baseline;^b ^Receipt of guidelines by mail;^c ^Assessment of control group during first period of study, followed by intervention and assessment of experimental group;^d ^Rost et al. (2001): comparison recently treated patients vs. patients beginning new treatment episode, analysis for both intervention groups.

**Abbreviations: **CES-D - Center for Epidemiologic Studies Depression Scale; CIDI - Composite International Diagnostic Interview; GDS - Geriatric Depression Scale; GP - General Practitioner; HADS-D/A - Hospital Anxiety and Depression Scale; HAM-D - Hamilton Depression Scale; MD - Major Depression; MMSE - Mini Mental Status Examination; mo - month(s); PRIME-MD - Primary care Evaluation of Mental Disorders; QI - Quality Improvement; UC - Usual Care; w/- with; WL - Waiting List; yrs - years.

### Study characteristics

All, but one, studies were conducted in anglophone countries, among them Great Britain (n = 4) and one study each in Canada, the United States and Australia. The sample sizes varied from 145 [33] to 733 [34,35]. Three studies included patients with a categorical (e.g. diagnostic system based) diagnosis of depression [33,36,37], while the other five made use of symptom rating scales (self-report scales). Gask et al. (2004) and Worrall et al. (1999) both based their samples on referrals by the general practitioner (having the GP determine whether the patient was depressed) but applied dimensional instruments to ensure accuracy of diagnoses [38,39]. All but one study used a cluster allocation design, randomising all included general practices to either intervention or control group. Only Llewellyn-Jones et al. (1999) conducted a serial designed survey, randomising each consecutive patient to the experimental groups [40]. Four research groups planned to train the control group practitioners after the end of the trial while the other four had them assigned to usual care groups with no further support provided. However, three study teams chose to provide the physicians of the control groups with depression specific guidelines [36,39,40]. In the Dutch study it is highlighted, that all practitioners are generally encouraged to adhere to guideline concordant treatment [33].

### Interventions

As for interventional strategies, education and training of the participating general practitioners was the sole intervention in three studies [33,38,41]. These studies did not provide any other organisational support for the practices. King et al. (2002) pose an exception to the other studies, since physicians here are trained to provide a specific interventional method (brief cognitive behavioural therapy) [41], while in the remaining trial physicians were only provided with lectures on assessment and treatment of depression. Four studies made use of guideline implementation [35-37,39]. These studies can be seen as providing a more intense intervention, as practitioners were trained and additionally received guidelines and guideline explanations. Rost et al. (2000) and Worrall et al. (1999) focussed the GP training on implementing guidelines [37,39]. Thompson et al. (2000) also educated practitioners but additionally tried to implement guideline concordant treatment [35]. Baker et al. (2001) used a tailored application of practitioner training by firstly analysing possible obstacles for successful guidelines implementation and then delivering individualised help to the GPs [36].

Regarding more complex interventions, it can be concluded that provider education plays the central part in the programme conducted by Llewellyn-Jones et al. (1999).

The mean quality score of all studies was at 34.91 points and ranged from 26 to 39 (individual scores in table 2). Criteria such as random allocation as proposed by the Cochrane Collaboration Handbook were adequately addressed by all studies [42].

### Effectiveness of provider training

Table 3 summarises all study results. The three studies solely providing physician education found no change in symptom severity. Neither lectures for more qualified assessment and treatment [33,38], nor training in brief cognitive behavioural therapy [41] led to significant symptom change in patients of trained physicians. Introducing additional guidelines and using them during practitioner training, two studies showed a mid and long term significant change in symptom load [39,43-45]. Both trials report small effect sizes (d = 0.22-0.29). Short term, one study was able to show an increases probability of reducing the depression score below a clinically relevant cut-point [36]. Yet, another study fails to show effects of the intervention introducing guidelines. Not only was there no effect of the practitioner training on diagnosis sensitivity and specificity, patient also do not profit symptom wise or regarding hospital admittance [34,35].

**Table 3:** Study Results

Study	Follow Up	Attrition Rate %^a^	Outcome	Results	Limitations	Effect Size^b^
Baker (2001) [36]	16 weeks	6	Proportion of patients w/BDI < 11	Sign. diff in proportion of patients w/BDI > 11 (OR = 2.5)	Tailored intervention that makes GP comparison impossible since they all received diff kinds of intervention	/

Bosmans (2006) [33]	12 mo	21	PRIME-MD	No sign. diff in MD recovery	Possible Hawthorne effect, less severe episodes of MD in primary care, no blinding of patients	-0.07

Gask (2004) [38]	3, 12 mo	37	HAM-D	No sign. diff in scores at both follow up points	Use of a new-onset (depressed less than 6 mo) sample Attrition rate rather high	-0.24

Kendrick (2001) [34]	12 mo	19	Hospital Admittance	No sign. difference	Implemented guidelines had been tested in highly selected samples Dimensional diagnosis Potential conservative bias (chronic depressed patients)	/

Thompson (2000) [35]	6 weeks/6 mo	50	HAD	No diff in improvement, no diff in caseness rating at both points Only patients recognized as cases at baseline improve sign. during first 6 weeks (p = 0.044), no diff at 6 mo	See Kendrick (2001)	/

			Diagnosis sensitivity	No diff in sensitivity nor specificity	See Kendrick (2001)	/

King (2002) [41]	6 mo	10	BDI	No sign. diff in BDI scores (p = 0.84)	Smaller sample than anticipated Cut off score for inclusion rather high (makes intervention effect of CBT by lay GPs less likely)	0.08

Llewellyn-Jones (1999) [40]	9.5 mo	23	GDS	Sign. change in GDS scores	Serial mono-centered design Control group assessment before implementation of intervention	-0.17

Rost (2001) [44]	6 mo	10	mCES-D	Sign. reduction in score in patients beginning new treatment episode	GP training effect unclear, feedback of diagnosis could be responsible for treatment effect Homogenous sample	-0.29

Pyne (2003) [43]	12 mo	65	Depression severity	Sign. decrease (7.7 units) in experimental group	See Rost (2001)	/

Rost (2005) [45]	24 mo	70	Depression Free Days	Sign. more depression free days in experimental group (647.6 vs. 558.2)	See Rost (2001)	/

Worrall (1999) [39]	6 mo	?	Gain score CES-D	Sign. improvement in experimental group	Possible Hawthorne effect	-0.22

^a ^all groups, last follow up;^b ^calculated as the mean difference of experimental and control group at the latest post-treatment measurement;^c ^adopted from Bower et al. (2006) [45].

**Abbreviations: **BDI - Beck's Depression Inventory; CBT - Cognitive Behavioral Therapy; CE Ratio - Cost Effectiveness Ratio; mCES-D - (modified) Center for Epidemiologic Studies Depression Scale; CIDI - Composite International Diagnostic Interview; diff - difference; GDS - Geriatric Depression Scale; GP - General Practitioner; HAD - Hospital Anxiety and Depression Scale; HAM-D Hamilton Depression Scale; mo - month(s); MD - Major Depression; PRIME-MD - Primary care Evaluation of Mental Disorders; QALY - Quality of Adjusted Life Year; SF12 - Short Form 12-Item Health Survey; sign - significant

As for the more complex interventional strategies, there is one study in which general practitioner and provider training plays a central role [40]. In a sample of elderly (65+) adults in a residential facility, Llewellyn-Jones and colleagues (1999) show a significant change in symptom quantity associated with a small effect size of Cohen's d = 0.17.

In studies that provided guidelines for the non-trained control group practitioners [36,39,40], additional training and interventions in the experimental groups led to positive outcome changes (see tables 2 and 3).

### Meta-analysis

**Figure 2:**
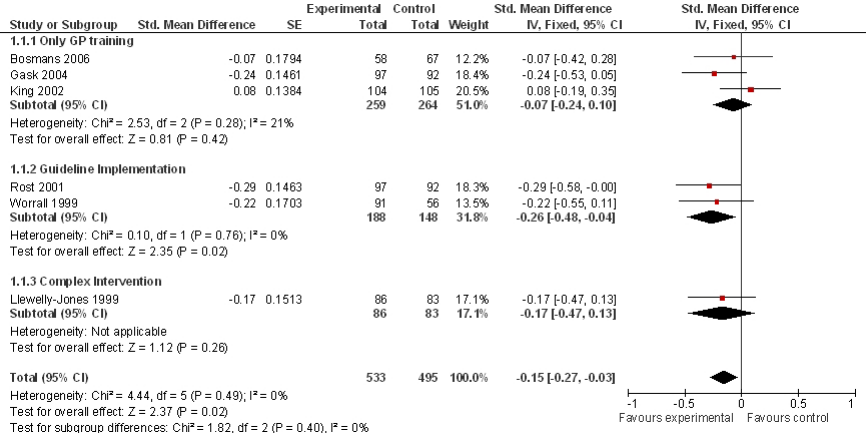
**Forest-Plot**.

The forest-plot of the conducted meta-analysis can be found in Figure 2. Three studies with only provider training provided data for meta-analysis. They found a non-significant decrease in depressive symptom load (pooled effect size d=-0.07, 95% CI -0.24 to 0.10, I^2 ^= 21%).

Two studies were categorized as implementing additional guidelines to primary care. They showed the highest pooled effect sizes and a significant decrease in depressive symptoms in the intervention groups (d=-0.26, 95% CI -0.48 to -0.04). The overall effect size, including a study with a more complex intervention was determined at d=-0.15 (95% CI -0.27 to -0.03]), favouring the experimental groups.

## Discussion

It is apparent that there are only few trials evaluating the effect of primary care physician education on health outcomes of patients. Especially during the last six years no results of education based interventions have been published. It seems that the research focus has shifted to more complex interventions encompassing collaborative and stepped care approaches by introducing mental health specialists to the primary care setting [46-48]. Regarding the results of the reviewed studies, this approach seems more than justified - it has yet to be shown that training practitioners alone yields significant symptom changes; however, this conclusion is only based on three relevant studies that themselves are highly diverse. While the study by Gask et al. (2004) struggles with high attrition rates, King et al. (2002) used a rather high cut-off and included chronically depressed patients, possibly leading to a conservative bias and therefore underestimating the treatment effect [38,41]. The authors argue that the applied kind of brief cognitive behavioural therapy might have been treatment approach not sufficiently intense for highly depressed patients. Bosmans et al. (2006) find that including less severely affected patients might have led to an underestimation of the efficacy of anti-depressant treatment [33].

Sample selection plays a major role in assessing treatment effects in general. One could assume that severely affected patients benefit more from treatment in clinical studies (as they can show a higher reduction in quantity of symptoms). In line with this, a categorical diagnostic approach for patient inclusion by applying diagnostic categories as provided by the Diagnostic and Statistical Manual of Mental Disorders (DSM) might lead to a sample of more severely affected patients [33,36,37].

Furthermore, the kind of treatment has an effect. In the context of stepped care, this issue is addressed by providing more-intense treatment options to higher affected patients [25].

Both argumentations can provide explanations for the positive results found by studies implementing additional guideline usage by general practitioners. Small effect sizes were shown by those studies including patients with new-onset depression, rather than chronically depressed patients (as done by Kendrick et al., 2001 not resulting in positive symptom change). Obviously, the effect of mere attention to the trained practitioners as well as to the patients themselves (referred to as Hawthorne effect) has to be considered a possible moderating variable in study designs. This would lead to better outcomes and performances of the control groups even though they received no active intervention; thus, the differences found may possibly be even higher.

The justification for more complex interventions to improve primary care depression treatment is replicated in the analysis of included studies and basically goes in line with a previous review [21], however, we did find more evidence in newer studies that support guideline implementation to be effective in symptom reduction. One trial applying more complex strategies both yielded significant changes in symptom outcomes; however, it remains unclear how much of the effect can be attributed to the physicians' education. Bower et al. (2006) conducted a meta-regression to evaluate active ingredients of collaborative care interventions [49]. In this analysis, primary care physician training is not associated with a positive change in depressive symptoms nor with a change in antidepressant use even in univariate calculations. Rather than provider education, systematic identification of patients, professional background of staff and supervision proved to be significant predictors of symptom change. It becomes clear that researchers should not assume an additive effect of treatment modules; especially in view of economic considerations, collaborative care cannot mean implementing as many treatment options as possible. This analysis of one specific potentially effective part of collaborative care is leading the way to a more thorough understanding of complex interventions and has to be pursued without neglecting the fact that more simple interventions can also lead to significant changes in patient outcomes as shown in this review.

However, it may not be appropriate to solely focus on outcomes of symptomatology as enhanced primary care supply may not be directly associated with such. Even the included studies show a rise in adherence to medication treatment [39] and medication treatment in general [34,44,50]. It has been shown that effectiveness of antidepressant treatment increases with depression severity [51]; an effect of increased antidepressant treatment in a sample of mildly depressed patients will therefore be small [as seen in the studies by [33,39]].

### Strengths and Limitations

This review only included randomised controlled trials, and therefore neglected observational and non-randomised studies. RCTs often adhere to strict exclusion criteria, thus making generalisability to primary care patients difficult. This also applies to the current review since studies with specialised co-morbid patient groups were excluded; however, regarding the heterogeneity of primary care patients, an adequate representation of studies seems hard to achieve in any case. The reported studies differ substantially in content, duration, intensity and frequency of the intervention programmes, making comparisons difficult. However, we were able to conduct a meta-analysis, quantifying the results of the studies. Meta-regression that could help determine the influence of these factors was not applicable due to the limited number of studies.

This partly results from the relatively narrow search strategy; only when education or training efforts of GPs were mentioned within title and abstract, the article was found with the applied search strategy. Earlier publications (before 1999) were not searched. Gilbody et al. mention one previous trial that showed positive effects of provider training but equally emphasise methodological weakness of this trial [21,52], so we did include relevant trials that live up to methodological requirements.".

Furthermore, a possible publication bias cannot be ruled out or determined with a qualitative review as this, especially under the regard of not searching grey literature. Regarding the fact that almost only studies from anglophone countries were found might be an indicator for unpublished studies with negative outcomes from other countries.

## Conclusions

It seems that provider training, if combined with guideline implementation, contributes to enhanced care for depression in primary care even associated with possible positive symptom changes. Providing a guideline and training practitioners to adhere to guideline-concordant treatment might be a measure of intervention that endures even after the intervention ends.

## Competing interests

The authors declare that they have no competing interests.

## Authors' contributions

CS, ML and SRH outlined and specified the research questions. The principal author and ML conducted the literature search and screened abstracts and titles. Article inclusion and study quality was also evaluated by ML and SRH. CS wrote the first draft of the manuscript. HHK and HvdB revised it critically for important intellectual content. All authors contributed to and have approved the final manuscript.

## Pre-publication history

The pre-publication history for this paper can be accessed here:

http://www.biomedcentral.com/1472-6963/12/10/prepub

## Supplementary Material

Additional file 1 **Search terms for Medline**. Details on the search strategy for Medline. Click here for file

Additional file 2 **List and references of excluded studies**. Overview of reason for exclusion. Click here for file
