# Supplementary material for: Does GP training in depression care affect patient outcome? - A systematic review and meta-analysis
Source: BMC Health Serv Res. 2012 Jan 10;12:10. doi: 10.1186/1472-6963-12-10 (PMC3266633; doi:10.1186/1472-6963-12-10)
Supplement: Additional file 1 — Search terms for Medline. Details on the search strategy for Medline. [file 1472-6963-12-10-S1.DOC]

Additional File 1 Search terms for Medline

("depressive disorder"[MeSH Terms] OR ("depressive"[All Fields] AND "disorder"[All Fields]) OR "depressive disorder"[All Fields] OR "depression"[All Fields] OR "depression"[MeSH Terms]) AND ("general practice"[All Fields] OR "general practitioner"[All Fields] OR "primary care"[All Fields] OR family practice)) AND (education OR training) AND (English[lang] OR German[lang])
