# Supplementary material for: Does GP training in depression care affect patient outcome? - A systematic review and meta-analysis
Source: BMC Health Serv Res. 2012 Jan 10;12:10. doi: 10.1186/1472-6963-12-10 (PMC3266633; doi:10.1186/1472-6963-12-10)
Supplement: Additional file 2 — List and references of excluded studies. Overview of reason for exclusion. [file 1472-6963-12-10-S2.DOC]

**Additional File 2: List of excluded studies**

| **Author** | **Year** | **Exclusion criteria** |
| --- | --- | --- |
| Brown et al. | 2000 | GP training not part of intervention |
| Bruce et al. | 2004 | No control group |
| Coleman et al. | 1999 | Focus not on depression care |
| Dietrich et al. | 2004 | No control group |
| Dobscha et al. | 2006 | GP training not part of intervention |
| Gerrity et al. | 1999 | No relevant outcomes |
| Hegel et al. | 2004 | No relevant outcomes |
| Katon et al. | 1999 | GP training not part of intervention |
| Katzelnick et al. | 2000 | Specific study sample |
| Lin et al. | 2001 | No relevant outcomes |
| Liu et al. | 2003 | Collaborative Care |
| Mihalopoulos, et al. | 2005 | No relevant outcomes |
| Mynors-Wallis et al. | 2000 | GP training not part of intervention |
| Rubenstein et al. | 1999 | No relevant outcomes |
| Schoenbaum et al. | 2001 | Collaborative Care |
| Sherbourne et al. | 2001 | Collaborative Care |
| Simon et al. | 2000 | GP training not part of intervention |
| Simon et al. | 2001 | Specific study sample |
| Tiemens et al. | 1999 | No RCT |
| Unützer et al. | 2001 | No relevant outcomes |
| Unützer et al. | 2002 | GP training not part of intervention |
| van Os et al. | 1999 | No RCT |
| Van Os et al. | 2004 | No RCT |
| Wells et al. | 2000 | Collaborative Care |
| Wells et al. | 2004 | No relevant outcomes |
| Williams et al. | 1999 | GP training not part of intervention |

GP – General Practitioner, RCT – Randomised Controlled Trial.
